# Blood Serum Therapeutics

**Published:** 1894-11

**Authors:** 


### EDITORIAL.

### BLOOD SERUM THERAPEUTICS.

Recent discoveries in the curative action of the blood serum of animals immunized by successive inoculations of cultures of the organisms of an infectious disease, when applied to cases of fresh infection in persons, is the greatest triumph of medical science in this age. It must be an unanswerable argument in favor of the study of bacteriology to those so-called practical individuals who believe that there is no good in laboratory work. From the day of the discovery of the fact that all infectious diseases were due to the action of specific organisms upon the body, began the study of the true science of medicine. Upon the study of bacteriology depends the rational therapeutics of future medicine.

